# Lung Resection for Chronic Pulmonary Aspergillosis With an Infected Bulla

**DOI:** 10.1002/ccr3.71274

**Published:** 2025-10-14

**Authors:** Kasumi Tamagawa, Shohei Mori, Yu Suyama, Makoto Odaka, Naoki Toya, Takashi Ohtsuka

**Affiliations:** ^1^ Department of Surgery The Jikei University Kashiwa Hospital Chiba Japan; ^2^ Division of Thoracic Surgery, Department of Surgery The Jikei University School of Medicine Tokyo Japan

**Keywords:** bulla, lung pulmonary aspergillosis, surgery

## Abstract

Lung resection for chronic pulmonary aspergillosis with infected bulla may unavoidably leave some infected tissue due to adhesions to the significant vessels and nerves; nevertheless, the treatment strategy of resection for most of the lesion and pre‐ and postoperative antifungal medication can be expected to be effective and even curative.

## Graphical abstract

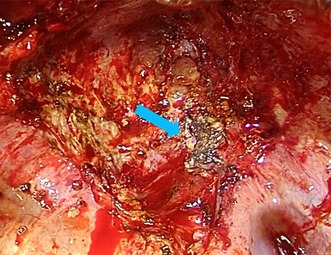

## 1 Introduction

Chronic pulmonary aspergillosis (CPA) is a disease in which *Aspergillus* chronically infects the lungs, causing inflammation and often disruption of lung tissue structure. CPA often occurs due to *Aspergillus* colonization and spoilage by a previous or current underlying lung disease (e.g., pulmonary tuberculosis, non‐tuberculosis mycobacterium infection, bronchiectasis, and pulmonary bulla) and may progress slowly and eventually be fatal if not treated appropriately or with refractoriness to treatment [1]. CPA includes various forms, such as single pulmonary aspergilloma (SA), chronic cavitary pulmonary aspergillosis, chronic fibrosing pulmonary aspergillosis, aspergillus nodule, and subacute invasive aspergillosis, which may overlap [1]. Surgical resection is the first‐line treatment for SA, while treatment for other CPA is based on long‐term antifungal medication [1]. However, in cases of intolerance/refractoriness to antifungal drugs or the presence of life‐threatening hemoptysis, surgical resection may be considered as a curative treatment [1]. The success of surgical resection depends on the ability to completely resect the lesion without the spillage of fungal components into the pleural cavity [2, 3].

Pulmonary bulla commonly develop in the lung apex and may be the underlying disease for CPA [4]. Therefore, difficulties are associated with completely resecting infected bulla in the lung apex caused by *Aspergillus* due to the possibility of severe adhesions to the surrounding significant vessels and nerves.

We herein describe a case of CPA with a pulmonary bulla, which was successfully treated with lung resection and antifungal medication for the residual infected bulla wall.

## 2 Case History/Examination

**FIGURE 1:**
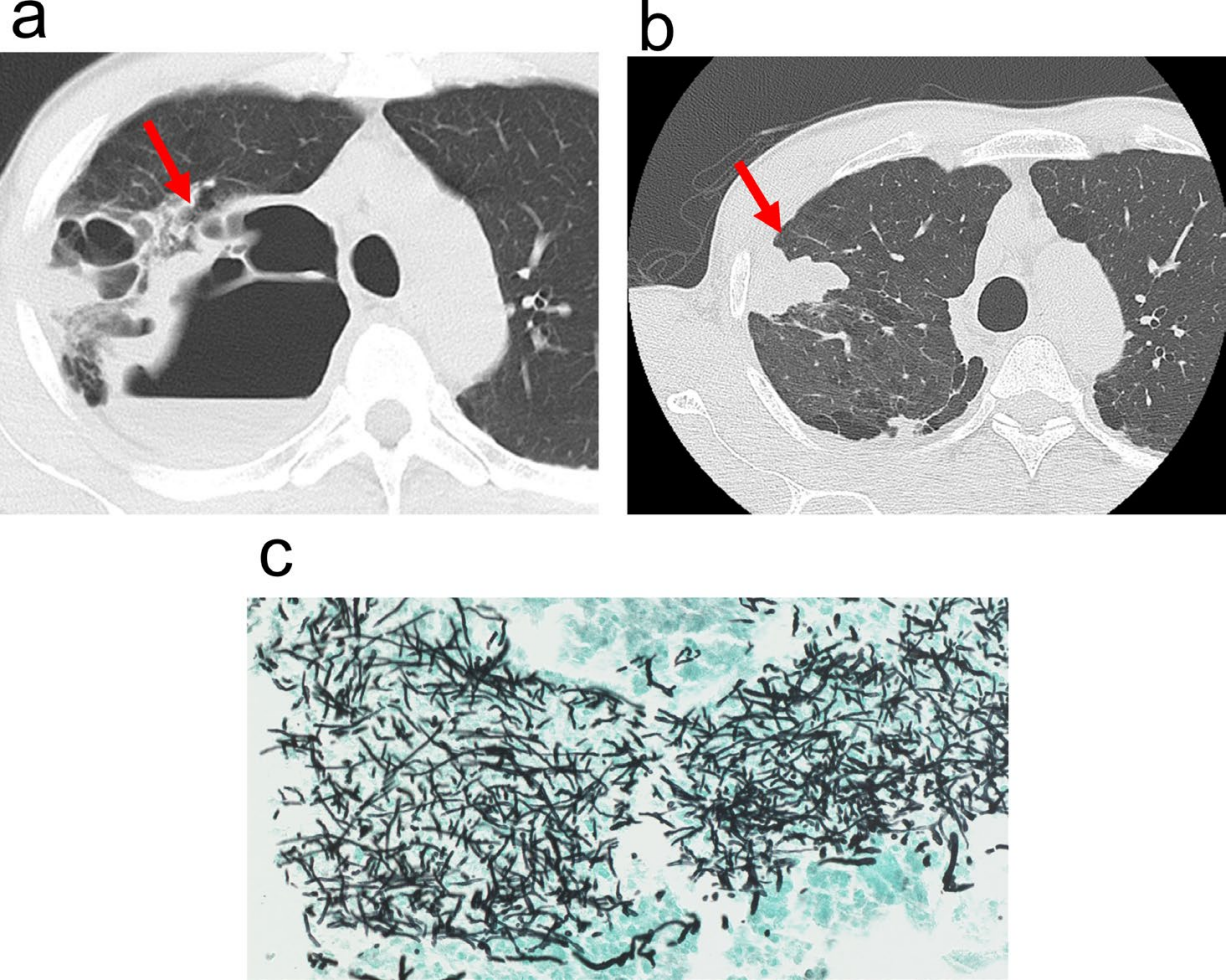
Preoperative findings. (a) An infected bulla with fluid collection (arrow) at the lung apex. (b) An irregularly shaped mass (arrow) in the right upper lobe. (c) A computed tomography‐guided needle biopsy specimen: Grocott's stain revealed many swarming Y‐branched septate hyphae.

A 57‐year‐old man presented with fever and right chest pain. Chest computed tomography (CT) showed a bulla in the right pulmonary apex with thickened walls and internal fluid collection, which was diagnosed as an infected bulla (Figure 1a). Symptoms were relieved by antimicrobial medication; however, subsequent CT revealed an irregularly shaped mass near the bulla (Figure 1b). The mass increased in size over time, and CT‐guided biopsy was performed. In the biopsy specimen, Grocott's stain revealed many swarming Y‐branched septate hyphae (Figure 1c). Laboratory data showed that slightly elevated serum C‐reactive protein and serum (1,3)‐β‐d‐glucan, 0.86 mg/dL and 12.6 pg/mL, respectively. Serum galactomannan antigen was negative and serum *Aspergillus* IgG was positive. Although the culture of the biopsy specimen was negative, we clinically diagnosed it as CPA.

## 3 Treatment

Voriconazole, 200 mg twice daily, as an antifungal medication, was started; however, CT 3 months later showed no regression of the mass shadow and increased fluid collection in the bulla. Since the patient was considered to have CPA refractory to antifungal medication, surgical lung resection was planned. The patient had sufficient pulmonary function and a good nutritional status.

**FIGURE 2:**
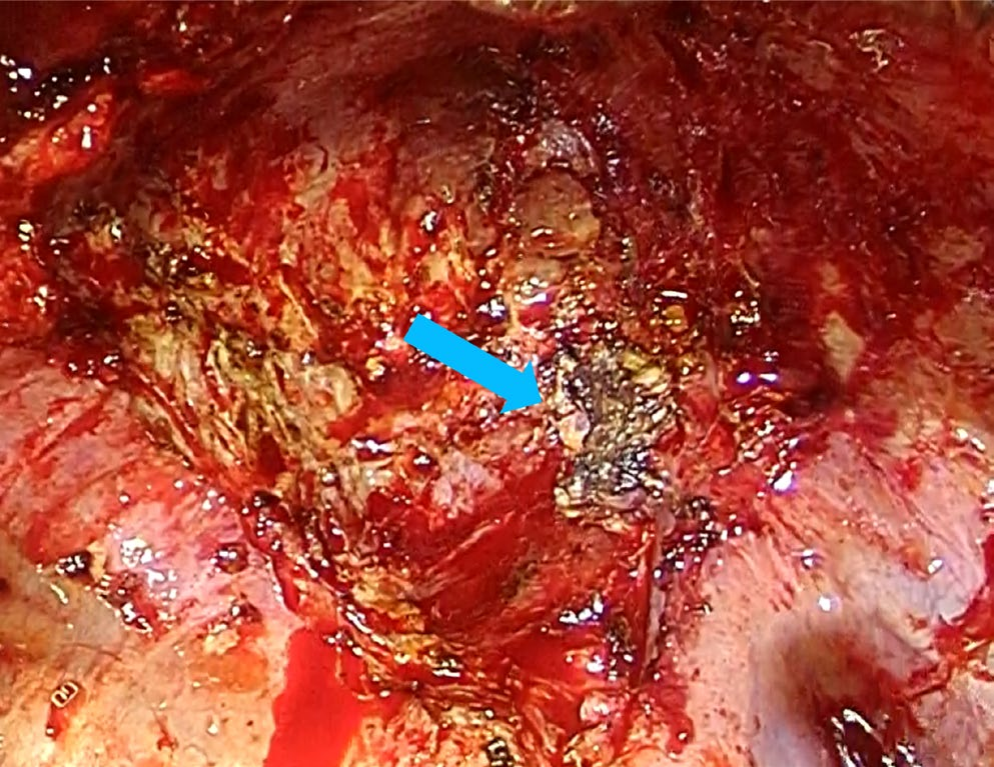
Intraoperative findings. Residual part of the bulla wall (arrow) that was inseparable from the right brachiocephalic vein.

Surgery was performed with thoracoscopic assistance through the fifth intercostal space thoracotomy (Video S1). Adhesions between the mass and chest wall were mild, whereas the bulla wall at the lung apex had severe adhesions to the chest wall and upper mediastinum. Due to unintentional intrusion into the bulla during dissection between the bulla and chest wall, the surgical plan was changed and upper lobectomy was preceded by the division of the bulla. Most of the remaining bulla wall was fragmented and removed; however, a part strongly adhered to the right brachiocephalic vein and was considered to be inseparable (Figure 2). The residual bulla wall was cauterized with soft coagulation and the thoracic cavity was irrigated with a large volume of warm saline. The upper lobe bronchial stump was covered with an intercostal muscle flap.

**FIGURE 3:**
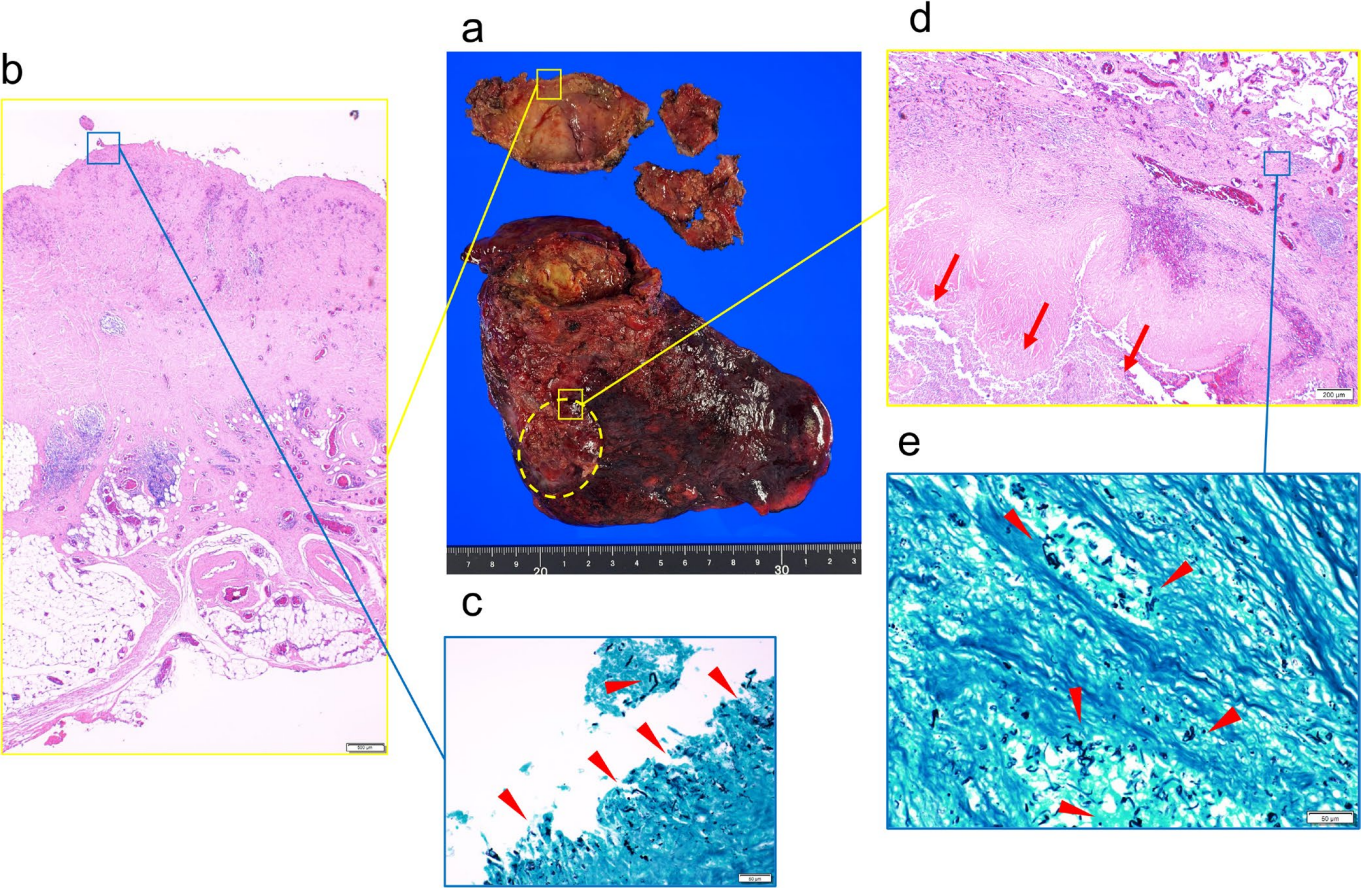
Pathological findings. (a) Surgically resected specimens were fragmented bulla walls and the right upper lobe including the lower half of the bulla and a 5‐cm mass (dotted line circle) was detected at the caudal side of the bulla, predominantly in S^2^. (b, c) Microscopic findings of the bulla wall revealed the presence of septate hyphae (arrow heads) inside the bulla wall. (d, e) Microscopic findings of the mass revealed destruction of the surrounding alveolar structures with fungal infiltration (arrow heads) and internal necrosis of the mass.

Figure 3a shows surgically resected specimens that were fragmented bulla walls and the right upper lobe, including the lower half of the bulla, and a 5‐cm mass was detected at the caudal side of the bulla, predominantly in S^2^. Microscopic findings of the bulla wall revealed the presence of septate hyphae inside the bulla wall (Figure 3b,c), and microscopic findings of the mass revealed destruction of the surrounding alveolar structures with fungal infiltration and internal necrosis of the mass (Figure 3d,e).

## 4 Outcome and Follow‐Up

The patient was discharged on postoperative day 22 without complications.

According to the recommendation of postoperative antifungal medication in case of incomplete resection by the clinical guidelines for the management of CPA published in 2016, voriconazole was started as postoperative therapy in this case. Since the guidelines state that the duration of medication cannot be universally recommended and should be individualized, we finished the medication 1 year postoperatively because there was no evidence of recurrence.

**FIGURE 4:**
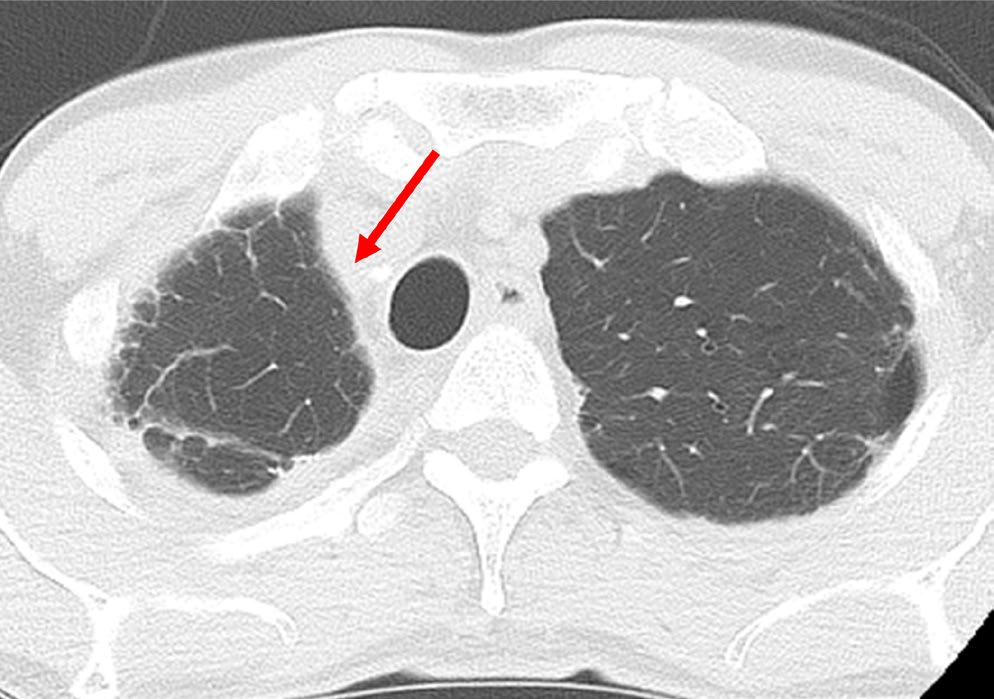
Postoperative findings. The preserved lung is expanding well (arrow) without dead space.

CT at 2 years postoperatively showed good residual lung expansion, no dead space, and no recurrence of CPA (Figure 4). Video S2 shows pre‐ and postoperative radiological images.

## 5 Discussion

This case of CPA with an infected bulla was successfully treated with lung resection and postoperative antifungal medication, although the bulla was unavoidably opened and there was residual infected tissue after resection due to severe adhesions to the right brachiocephalic vein.

Pulmonary bulla may be an underlying disease for CPA [1]. Chronic persistent infection of the bulla causes severe adhesions to the surrounding structures. Since the most common anatomical site of the bulla is the lung apex, it adheres to the surrounding significant vessels and nerves, such as the subclavian artery, subclavian vein, right brachiocephalic vein, superior vena cava, phrenic nerve, vagus nerve, and sympathetic nerve trunks [4]. Therefore, the surgical strategy selected needs to avoid damage to these vessels and nerves, and intraoperative decisions may result in residual infected tissue after resection. In the present case, as much of the bulla wall as possible was fragmented and removed; however, part of the wall strongly adhered to the right brachiocephalic vein and was inseparable from the vessel. Artificial vessel replacement needs to be avoided in surgery for infection.

The success of surgery for CPA is dependent on complete resection of the lesion and no spillage of the fungal component [2, 3]. However, complete resection is often difficult to achieve in practice. Therefore, the postoperative recurrence rate is higher in chronic cavitary pulmonary aspergillosis or chronic fibrosing pulmonary aspergillosis than in SA [5, 6]. Although the infected bulla was unavoidably opened and infected tissue remained after resection in the present case, possible reasons for the good outcome were as follows. The patient's background factors included non‐elderly, the absence of frailty, and a good nutritional status. Furthermore, preoperative antifungal medication, which has been identified as a protective factor against recurrence, was administered [7]. In addition, the lesion was confined to the right upper lobe, the pulmonary bulla as an underlying lung disease was also localized to the lung apex, and there were no adhesions to the middle or lower lobes. These conditions led to good expansion of the preserved lung, which eliminated dead space. Moreover, postoperative antifungal medication was performed for a sufficient duration, namely, 1 year [7].

Oral triazoles, including voriconazole and itraconazole, are considered the standard of care for CPA [1]. In this case, we selected voriconazole as pre‐ and postoperative antifungal medication, considering the efficacy and acceptable tolerability reported from several studies [8, 9].

There are a number of limitations that need to be addressed. Since the postoperative follow‐up was only 2 years, it may not have been adequate to confirm whether the patient was completely cured. Previous studies reported 80% of recurrence cases are within 3 years and a mean postoperative period of recurrence of 15 months [6, 7]. Furthermore, the good outcome in the present case may not be generalized to CPA with bulla as an underlying lung disease. It may be a comprehensive result of the multiple favorable factors described above. Regarding CPA from pulmonary bulla, a previous study demonstrated that the outcome of lung resection for CPA did not significantly differ between underlying lung diseases [10]. In addition, the recommended duration of postoperative antifungal medication in cases of incomplete resection has not yet been established [1]. We considered 1 year to be adequate because of the lack of radiological evidence of recurrence 1 year after surgery. However, since the continuation of medication for a longer term may be beneficial, each case needs to be individually assessed.

In conclusion, lung resection for CPA with infected bulla in the lung apex may unavoidably result in residual infected tissue due to adhesions to the surrounding significant vessels and nerves; nevertheless, the result does not mean treatment failure, and the treatment strategy of resection for most of the lesion and pre‐ and postoperative antifungal medication can be expected to be effective and even curative.

## Author Contributions

**Kasumi Tamagawa:** writing – original draft. **Shohei Mori:** investigation, writing – review and editing. **Yu Suyama:** writing – review and editing. **Makoto Odaka:** investigation, writing – review and editing. **Naoki Toya:** supervision, writing – review and editing. **Takashi Ohtsuka:** supervision, writing – review and editing.

## Consent

The patient provided signed authorization to publish the information disclosed in this report.

## Conflicts of Interest

The authors declare no conflicts of interest.

## Supporting information


**Video S1:** Surgical video.

**Video S2:** Pre‐ and postoperative radiological images.

## Data Availability

The authors have nothing to report.

## References

[1] D. W. Denning , J. Cadranel , C. Beigelman‐Aubry , et al., “Chronic Pulmonary Aspergillosis: Rationale and Clinical Guidelines for Diagnosis and Management,” European Respiratory Journal 47 (2016): 45–68. 26699723 10.1183/13993003.00583-2015

[2] A. Brik , A. M. Salem , A. R. Kamal , et al., “Surgical Outcome of Pulmonary Aspergilloma,” European Journal of Cardio‐Thoracic Surgery 34 (2008): 882–885. 18701313 10.1016/j.ejcts.2008.06.049

[3] S. Farid , S. Mohamed , M. Devbhandari , et al., “Results of Surgery for Chronic Pulmonary Aspergillosis, Optimal Antifungal Therapy and Proposed High Risk Factors for Recurrence—A National Centre's Experience,” Journal of Cardiothoracic Surgery 8 (2013): 180. 23915502 10.1186/1749-8090-8-180 PMC3750592

[4] A. R. Casha , A. Manché , R. Gatt , et al., “Is There a Biomechanical Cause for Spontaneous Pneumothorax?,” European Journal of Cardio‐Thoracic Surgery 45 (2014): 1011–1016. 24644314 10.1093/ejcts/ezt659

[5] M. M. El Hammoumi , O. Slaoui , F. El Oueriachi , and E. H. Kabiri , “Lung Resection in Pulmonary Aspergilloma: Experience of a Moroccan Center,” BMC Surgery 15 (2015): 114. 26475478 10.1186/s12893-015-0103-4 PMC4608121

[6] C. Shen , G. Qiao , C. Wang , F. Jin , and Y. Zhang , “Outcomes of Surgery for Different Types of Chronic Pulmonary Aspergillosis: Results From a Single‐Center, Retrospective Cohort Study,” BMC Pulmonary Medicine 22 (2022): 40. 35045860 10.1186/s12890-022-01836-z PMC8772183

[7] F. Setianingrum , R. Rautemaa‐Richardson , R. Shah , and D. W. Denning , “Clinical Outcomes of Patients With Chronic Pulmonary Aspergillosis Managed Surgically,” European Journal of Cardio‐Thoracic Surgery 58 (2020): 997–1003. 32386208 10.1093/ejcts/ezaa137

[8] J. Cadranel , B. Philippe , C. Hennequin , et al., “Voriconazole for Chronic Pulmonary Aspergillosis: A Prospective Multicenter Trial,” European Journal of Clinical Microbiology & Infectious Diseases 31 (2012): 3231–3239. 22782438 10.1007/s10096-012-1690-y PMC3479377

[9] T. Saito , S. Fujiuchi , Y. Tao , et al., “Efficacy and Safety of Voriconazole in the Treatment of Chronic Pulmonary Aspergillosis: Experience in Japan,” Infection 40 (2012): 661–667. 22956473 10.1007/s15010-012-0322-x

[10] A. Muniappan , L. F. Tapias , P. Butala , et al., “Surgical Therapy of Pulmonary Aspergillomas: A 30‐Year North American Experience,” Annals of Thoracic Surgery 97 (2014): 432–438. 24365218 10.1016/j.athoracsur.2013.10.050

